# Analysis of the heterogeneity of the immune microenvironment of bone metastases and new strategies of nanotechnology intervention

**DOI:** 10.3389/or.2026.1820639

**Published:** 2026-09-11

**Authors:** Chenyang Wu, Xueli Qiu, Chao Wang, Jinyu Bai, Xiaozhong Zhou

**Affiliations:** 1 Second Affiliated Hospital of Soochow University, Suzhou, China; 2 Suzhou Medical College of Soochow University, Suzhou, China

**Keywords:** bone metastasis, immune microenvironment, immunotherapy resistance, nanotechnology, targeted delivery

## Abstract

Bone metastases constitute one of the most severe complications in patients with advanced malignancies. Conventional treatment approaches face significant limitations: surgical intervention carries a high risk of recurrence; radiotherapy and chemotherapy often cause myelosuppression; and anti-resorptive agents provide only limited control over tumor progression. Although immunotherapy has achieved remarkable success in various solid tumors in recent years, its therapeutic impact on bone metastases remains limited. This limitation is largely attributed to the distinct immunosuppressive microenvironment within bone lesions, which is marked by (1) a substantial decline in both the quantity and functional activity of effector immune cells, coupled with an abnormal accumulation of immunosuppressive cell populations, and (2) the formation of a vicious cycle of “tumor growth-bone metabolic imbalance-immune suppression,” driven by complex signaling interactions among osteoblasts, osteoclasts, and tumor cells. Recently, nanomaterials have emerged as promising therapeutic platforms due to their precise targeting and delivery capacity, inherent immune-stimulatory properties, and potential to restore bone homeostasis. Nonetheless, translating nanoplatforms into clinical practice for skeletal lesions remains a formidable challenge. This review highlights how specialized organic-inorganic hybrids and biomimetic nanoplatforms have achieved the most significant microenvironment remodeling and osteolytic inhibition within intraosseous murine models. Ultimately, we provide an actionable roadmap detailing how integrating multi-omics precision profiles and temporal programmable delivery can bridge the gap between preclinical nanomedicine and clinical oncological translation.

## 1 Introduction

Bone metastasis represents one of the most critical complications in patients with advanced malignancies (1, 2). It refers to the pathological process in which primary tumor cells disseminate through the bloodstream or lymphatic system and establish secondary tumor sites within bone tissue (3). Epidemiological studies show that approximately 70% of patients with advanced breast or prostate cancer, and 30%–40% of those with advanced lung cancer, develop bone metastases (4–6). These metastases often induce osteolysis and are accompanied by severe bone pain, substantially impairing patients’ daily activities, sleep quality, and overall wellbeing, while also predisposing them to pathological fractures (7). Such fractures not only cause debilitating pain but also lead to impaired mobility, increasing the risk of long-term complications associated with prolonged immobility and bed rest (2). In addition, bone metastases may trigger hypercalcemia, as excessive osteoclastic activity releases large amounts of calcium into circulation; in severe cases, this metabolic disturbance can be life-threatening (8). Consequently, bone metastasis not only drastically reduces quality of life but also significantly contributes to higher mortality rates among affected patients (1).

The clinical management of bone metastases currently includes surgical resection, anti-resorptive agents, chemotherapy, and radiotherapy. However, each of these modalities faces substantial limitations: (1) Surgical intervention is generally restricted to patients with solitary bone lesions and carries a risk of tumor recurrence following resection (8, 9). (2) Anti-resorptive agents such as bisphosphonates and denosumab can alleviate bone pain and reduce the incidence of fractures, yet they exert no direct cytotoxic effect on tumor cells; moreover, prolonged use may result in serious complications, including osteonecrosis of the jaw (10–12). (3) Radiotherapy provides effective local disease control but is often complicated by myelosuppression (13–15). (4) Chemotherapy reduces systemic tumor burden but is similarly myelotoxic, frequently exacerbating leukopenia when combined with radiotherapy and causing treatment interruptions; its delivery to bone lesions is further hindered by the protective blood-bone barrier (16, 17). The advent of immunotherapy has introduced new optimism by harnessing the host immune system to recognize, attack, and eliminate tumor cells, thereby prolonging survival and improving quality of life in patients with primary tumors (18). Nevertheless, despite its transformative success in many solid cancers, immunotherapy has demonstrated limited efficacy in bone metastases (19). This poor response is primarily attributed to the unique immunosuppressive bone microenvironment, characterized by (1) a pronounced depletion of effector immune cells alongside abnormal accumulation of immunosuppressive populations, and (2) a complex signaling network among osteoblasts, osteoclasts, and tumor cells that perpetuates a vicious cycle of “tumor growth-bone metabolic imbalance-immune suppression” (20, 21).

In response to the persistent challenges in treating bone metastases, recent research has increasingly emphasized nanomaterial-based delivery systems in combination with established therapeutic modalities. These nanocarriers exhibit excellent targeting specificity, versatile functionalization and modification potential, and favorable biocompatibility (22, 23). By co-loading small-molecule agents, chemotherapeutics, or immunomodulatory drugs, nanomaterials can achieve tumor site-specific delivery while simultaneously activating the immune system and regulating bone homeostasis (24, 25). Such multifunctional therapeutic strategies hold significant promise for enhancing clinical outcomes in patients with bone metastases.

Although numerous investigations have explored either the immune microenvironment of bone metastases or the development of nanodrug delivery systems, comprehensive comparative analyses examining how distinct nanoplatforms modulate the tumor microenvironment remain scarce. To address this gap, this review adopts a cell-centric perspective to analyze the heterogeneous immune landscape of bone metastases and elucidate the mechanisms underlying the limited efficacy of current therapeutic interventions. We further evaluate recent progress in nanocarrier development, ranging from organic polymers and biomimetic vectors to organic-inorganic hybrid systems, and highlight their strategies for microenvironmental remodeling, including improved targeting precision and enhanced synergistic effects, to optimize treatment outcomes in bone metastatic disease. Finally, we discuss existing challenges and propose future directions, offering theoretical insights and technical perspectives to inform the advancement of immunotherapeutic approaches for patients with bone metastases.

## 2 Characteristics of the immune microenvironment in bone metastasis

**FIGURE 1:**
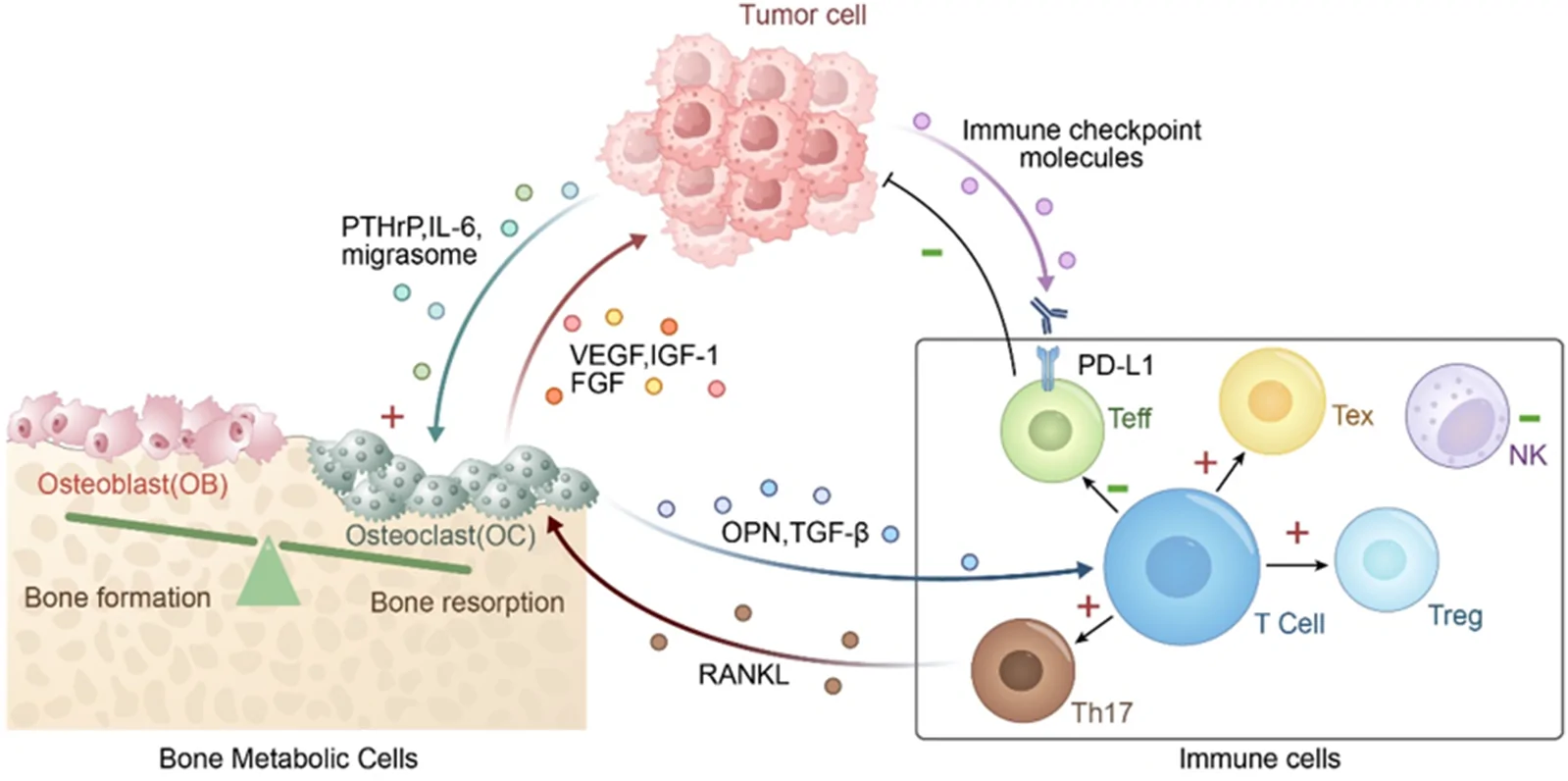
The interaction between bone, the immune system, and cancer cells. After tumor cells enter the bone marrow, they secrete PD-L1, PTHrP, IL-6, and migrasomes; these factors increase the RANKL/OPG ratio in osteoblasts and, via the RANKL pathway, promote osteoblast differentiation into osteoclast precursors and their eventual maturation into osteoclasts, causing bone resorption and bone destruction. Concurrently, tumors induce T cell exhaustion through immune checkpoints and stimulate stromal and immune cells to release chemokines, recruiting MDSCs and other immunosuppressive cells, thereby further amplifying osteolytic and immunosuppressive signaling. Activated osteoclasts secrete immunosuppressive molecules such as TGF-β, OPN, IL-6, and IDO1, which exhaust effector T/NK cells and drive T cell polarization toward Treg and Th17 phenotypes—Th17 cells secrete IL-17 to promote osteoclast differentiation, while Tregs suppress effector responses via pathways including CTLA-4—together producing an immune-cold microenvironment in bone metastases. In addition, growth factors released from bone matrix degradation reciprocally promote tumor growth, forming a malignant cycle of “tumor growth—bone metabolic imbalance—immune suppression.”

The immune microenvironment of bone metastases constitutes a highly complex and dynamically evolving pathological ecosystem (21, 26) (Figure 1). Its defining feature is profound immunosuppression, which provides a critical foundation for tumor cell colonization, proliferation, and evasion of immune surveillance within bone tissue (21, 27). Unlike that of primary tumors or other metastatic sites, the immune landscape of bone metastases displays distinctive compositional and functional characteristics (28, 29). Specifically, it is shaped by an abnormal enrichment of immunosuppressive cell populations, dysfunctional stromal elements, intricate networks of inhibitory soluble mediators, and unique physicochemical conditions (26, 27, 30, 31). Collectively, these factors generate a dynamic and profoundly immunosuppressive niche. More importantly, they do not act independently but rather engage in complex positive feedback interactions that synergistically suppress antitumor immunity, drive resistance to immunotherapy, and ultimately facilitate tumor progression and bone destruction (32, 33).

### 2.1 Tumor cells

The colonization of tumor cells within bone represents the initiating step of bone metastasis. After entering the bone via the bloodstream or lymphatic system, tumor cells secrete a variety of factors, including immune checkpoint molecules, diverse signaling mediators, and migrasomes (34–36). Immune checkpoint molecules such as PD-L1 can directly induce T-cell exhaustion through the PD-1/PD-L1 pathway (37). Meanwhile, traditional tumor-derived signaling molecules, such as PTHrP and IL-6, disrupt the physiological balance between bone formation and resorption by modulating osteoblast and osteoclast activity (38, 39). Notably, Lin et al. demonstrated that tumor-derived exosomes enriched with tumor-associated RNA establish spatiotemporal “tumor-osteoclast” interactions during the early phases of bone metastasis, thereby markedly enhancing osteoclast-mediated bone resorption (34). This bone degradation releases growth factors stored in the bone matrix, including insulin-like growth factor 1 (IGF-1), fibroblast growth factor (FGF), and vascular endothelial growth factor (VEGF), which subsequently promote tumor cell survival and proliferation, fueling a self-perpetuating “tumor growth-bone destruction” feedback loop that accelerates disease progression (40–42). In addition, activated osteoclasts secrete molecules such as OPN, TGF-β, and IL-6, which drive the differentiation of T cells into immunosuppressive subsets, thereby depleting effector immune cells capable of eliminating tumor cells and diminishing the response to immunotherapy (32, 43). Collectively, these direct and indirect mechanisms enable tumor cells to suppress immune activity within the bone microenvironment while simultaneously stimulating osteoclast-driven bone destruction, ultimately creating a vicious cycle of “tumor growth-bone destruction-immunosuppression” that poses major obstacles to the effective clinical management of bone metastasis.

### 2.2 Immune cells

In contrast to the proinflammatory microenvironment of primary tumors, the bone metastasis microenvironment is characterized by pronounced immunosuppressive features (28, 44). This is reflected in both the reduced proportion and functional impairment of key immune cell subsets responsible for antitumor activity, alongside a concomitant increase in the prevalence of ineffective or immunosuppressive cell populations that hinder tumor clearance (44, 45).

#### 2.2.1 T cell

T cells are central regulators of antitumor immunity, and both their abundance and functional integrity critically determine immune effectiveness (46, 47). Evidence indicates that, compared with primary tumors, bone metastases exhibit markedly reduced proportions of key effector T cells (Teffs), precursor exhausted T cells (Tpexs), memory T cells (Tms), and other subsets essential for sustaining tumor immunity (32). In contrast, the prevalence of immunosuppressive T-cell populations, such as regulatory T (Treg) cells and follicular helper T (Tfh) cells, is significantly elevated in the bone metastatic microenvironment (32, 45) (Table 1). This imbalance, characterized by the depletion of protective T-cell subsets alongside the enrichment of suppressive ones, contributes directly to the poor responsiveness of bone metastases to immunotherapy. The following section discusses in detail the quantitative and functional alterations of these T-cell subsets within the bone metastatic niche.

**TABLE 1:** Proportion of T cell subsets in the literature review.

T-cell type		Carcinoma *in situ*	Bone metastasis
CD4^+^Tcells	Tregs	20%–30%	25%–50%
CD8^,^T cells	Teff	10%–30%	An elevated frequency of exhausted CD8^+^ T cells has been observed; however, the exact proportion remains unreported
	Tex^+^Tpex	15%–40%	
γδT cells		1%–10%	No reports yet

Treg, regulatory T cell; Teff, effector T cell; Tex, exhausted T cell; Tpex, precursor exhausted T cell.

##### 2.2.1.1 CD8^+^ T cells

CD8^+^ T cells are the principal effector cells in antitumor immunity and can be subdivided into several functionally distinct subsets within the bone metastatic microenvironment, including Teff, Tpex, exhausted T cells (Tex), Tms, and tissue-resident memory (TRM) cells (48–50). Teff cells recognize tumor-associated antigens presented via MHC-I molecules and exert direct cytotoxicity through perforin, granzyme B, IFN-γ, TNF-α, and related mediators (51). However, in bone metastases, their activity is markedly suppressed by the high expression of inhibitory receptors such as PD-1 and CTLA-4, as well as by the immunosuppressive actions of Treg and MDSC populations (52, 53). Tpex cells, characterized by high TCF-1 expression and low checkpoint receptor levels, retain strong self-renewal capacity and function as a critical reservoir for sustaining antitumor responses (54). Yet, in bone metastases, aberrant Notch activation and macrophage-driven arginine depletion accelerate their transition toward terminal exhaustion (49). Tex cells display profound functional impairment and express elevated levels of checkpoint molecules including PD-1, TIM-3, LAG-3, and TOX (55). Although checkpoint blockade can partially restore Tex function at early stages, persistent exposure to immunosuppressive factors such as adenosine, RANKL, and IL-10 imposes deep transcriptional programming, sharply reducing their cytotoxicity and proliferative potential (56). Tm subsets, including central (Tcm) and effector memory (Tem) cells, rely on IL-7R/BCL-2 signaling to maintain long-term survival and rapid recall capacity; however, elements of the bone metastatic milieu, such as TGF-β, acidosis, and extracellular matrix (ECM) dysregulation, impede their infiltration and memory maintenance (57, 58). TRM cells, typically localized at the invasive margins of bone metastases, express CD69 and CD103, enabling continuous cytokine secretion and “bystander killing” of heterogeneous tumor populations (59). Nevertheless, in bone metastases, their migratory dynamics and metabolic fitness are compromised, likely due to ECM fibrosis, local hypoxia, and TGF-β-mediated repression (60, 61). Collectively, CD8^+^ T cells, despite their diverse subsets and unique antitumor potential, are functionally constrained in bone metastases by a multifaceted immunosuppressive network. This widespread dysfunction diminishes their cytotoxicity, persistence, and memory capacity, thereby facilitating immune evasion, accelerating tumor progression, and undermining the efficacy of immunotherapeutic strategies.

##### 2.2.1.2 CD4^+^ T cells

CD4^+^ T-cell subsets, including Th1, Th2, Th17, Treg cells, Tfh cells, and follicular Treg cells (Tfr), play distinct yet interrelated roles in shaping the immune microenvironment of bone metastases (53, 62). Th1 cells, characterized by T-bet expression and secretion of IFN-γ and TNF-α, activate macrophages, enhance CD8^+^ T-cell cytotoxicity, and inhibit osteoclast differentiation. However, their polarization depends on IL-12 and is frequently suppressed in bone metastases by TGF-β derived from PD-L2-inhibited Dendritic cells (DCs) and osteoblasts (57, 63, 64). By contrast, Th2 cells contribute prominently to immunosuppression through IL-4 and IL-13 secretion and polarize macrophages into an M2 phenotype (65). This Th2-M2 axis subsequently downregulates IL-12 while upregulating IL-10 and TGF-β, thereby impairing cytotoxic T lymphocytes (CTLs) and natural killer (NK) cells to facilitate immune escape within the metastatic niche (66). Th17 cells exert dual functions: on the one hand, IL-17A/F secretion promotes osteoblast antimicrobial peptide production and antitumor responses; on the other, RANKL-mediated signaling enhances osteoclast activity, while acidic conditions and IL-23 amplify this “double-edged sword” effect (67–69). Tregs, defined by FoxP3 and CD25 expression, suppress effector T cells through IL-10, IL-35, CTLA-4, and adenosine-A2AR signaling. Their expansion is further driven by tumor-derived exosomes, CD200/CD200R engagement, and hypoxic stress, making them central mediators of immune evasion in bone metastases (70, 71). Tfh and Tfr cells regulate B-cell responses via BCL-6, PD-1, and CTLA-4; however, their functions are reprogrammed in bone metastases by factors such as Galectin-9/TIM-3, BAFF, and IL-6-STAT3 signaling, leading to weakened or even pathogenic activity (72). Collectively, CD4^+^ T-cell subsets play multifaceted roles in the bone metastatic immune microenvironment, where their interactions are both synergistic and antagonistic. Yet, the dominance of immunosuppressive influences, including TGF-β, IL-10, acidic pH, exosomal signaling, and dysregulated pathways, disrupts the delicate balance among these subsets, establishing an immunosuppressive state that promotes tumor immune escape and disease progression.

##### 2.2.1.3 γδ T cells

γδT cells represent a unique subset of T lymphocytes. Unlike conventional αβT cells, they are distinguished by their ability to mount rapid responses to tumor antigen stimulation and enhance antitumor immunity through the secretion of IFN-γ and TNF-α (73, 74). Their antigen-independent cytotoxicity allows them to maintain immune surveillance even in tumor cells lacking MHC-I expression. Furthermore, γδT cells can directly induce tumor cell apoptosis and reduce tumor burden via TRAIL- and FasL-mediated pathways (75, 76). Despite their potential importance, the specific abundance and functional status of γδT cells within the bone metastatic microenvironment remain largely uncharacterized. Investigating their distribution and roles in this context may therefore provide novel therapeutic opportunities for bone metastatic disease.

To distill the complex landscape of T cell dysregulation within the bone metastatic niche, the relative abundance, functional phenotypes, and key molecular drivers of primary T cell subsets are systematically integrated. Exhausted CD8^+^ T (Tex) cells dominate the late-stage macro-metastases, characterized by marked depletion and high expression of terminal checkpoints (PD-1, TIM-3, LAG-3) driven by chronic TOX and T-bet transcriptional programming. Conversely, CD4^+^FoxP3^+^ regulatory T (Treg) cells are highly enriched from the early colonization phase, actively sustained by matrix-released TGF-β and tumor-derived CCL22, maintaining local immune tolerance. Furthermore, the balance between pathogenic IL-17-producing Th17 cells and γδT cells is severely disrupted by hyperactive osteoclastogenesis; the elevated RANKL and IL-23 levels in the niche polarize these subsets to accelerate both bone resorption and effector T-cell suppression. Rather than an encyclopedic enumeration of downstream signaling cascades, it is these core axes—specifically the TGF-β/SMAD3, RANK/RANKL, and STAT3/6 nodes—that represent the primary upstream check-points dictating T-cell fate and serving as viable targets for therapeutic nanomodulation.

In summary, T cells represent the primary effector population in antitumor immunity; however, within the unique microenvironment of bone metastases, their differentiation pathways are profoundly shaped by immunosuppressive influences. These conditions promote the expansion of suppressive subsets while diminishing protective populations, which contributes to the limited responsiveness of bone metastases to immunotherapy. Therefore, comprehensive studies examining the abundance, phenotypic heterogeneity, and functional states of T-cell subsets in the bone metastatic niche are essential for uncovering novel strategies to enhance therapeutic efficacy in patients with bone metastases.

#### 2.2.2 NK cells

NK cells are also crucial components of tumor immunity. They exert direct cytotoxic effects by releasing perforin and granzymes (e.g., perforin and GZMB) and secrete cytokines such as IFN-γ and TNF-α to recruit and activate other immune cells (77, 78). However, within the bone metastasis tumor microenvironment, tumor- and stromal cell-derived inhibitory mediators, including TGF-β, IL-10, PGE_2_, and adenosine, markedly suppress NK cell activity. The expression of activating surface receptors (such as NKG2D and DNAM-1) is diminished, while inhibitory receptors (including NKG2A and TIM-3) are upregulated, severely impairing NK cells’ ability to recognize and eliminate tumor cells (79, 80). Furthermore, tumor cells can shed soluble MICA/B, which binds to NKG2D as a decoy and further disables NK cell responses (81). Compounding these effects, a microenvironment characterized by elevated ROS, nutrient deprivation, and excessive lactate induces mitochondrial dysfunction and inhibits glycolysis in NK cells, depriving them of the metabolic energy required for cytotoxicity (82). Evidence also suggests that TME-driven metabolic impairments, such as inhibition of key enzymes like FBP1, further aggravate NK cell dysfunction (83). Collectively, these factors significantly reduce NK cell abundance and function in the bone metastatic niche, ultimately diminishing their tumor-killing capacity.

#### 2.2.3 Other immune cells

Other immunosuppressive cell populations within the bone metastasis microenvironment also display marked heterogeneity. Elevated expression of PD-L1 and IL-1β on tumor cell surfaces facilitates the differentiation of granulocytes into myeloid-derived suppressor cells (MDSCs). These MDSCs suppress T and NK cell activity, promote Treg expansion through the secretion of arginase, ROS, and immunosuppressive cytokines such as IL-10 and TGF-β, and simultaneously enhance osteoclast activity to accelerate bone destruction (32, 84, 85). In addition, tumor cells and osteoblasts promote M2 macrophage polarization via pathways including IL4R, MFGE8, Wnt, and CXCL14. Polarized M2 macrophages further inhibit CD8^+^ T cell function and stimulate Treg proliferation by releasing IL-10 and TGF-β (86). DCs are also affected, showing a 50%–70% decrease in infiltration within bone metastases and adopting a tolerogenic phenotype, characterized by the downregulation of MHC-II, CD80, and CD86, under the influence of the TGF-β/CXCL12 axis. These DCs differentiate into CD11b^+^ subsets that secrete IL-10, thereby driving Treg expansion and reinforcing multiple immune escape mechanisms (87, 88). Collectively, these immunosuppressive cell subsets predominantly differentiate toward regulatory phenotypes in the bone metastatic niche, further impairing the antitumor functions of T cells and NK cells.

#### 2.2.4 Structured variations of immune composition across tumor origins and metastatic stages

To transition from a purely qualitative assessment of immune heterogeneity to a structured biological framework, it is crucial to recognize that the immune composition of bone metastases varies predictably across primary tumor origins, histological types, and temporal stages of progression. Mechanistically, bone lesions originating from advanced breast cancer or non-small cell lung cancer (NSCLC) present a predominantly osteolytic phenotype, characterized by massive recruitment and hyperactivation of osteoclasts. This osteolytic microenvironment acts as a major reservoir for matrix-released cytokines, generating a profoundly dense, TGF-β-rich immunosuppressive niche characterized by high frequencies of CD4^+^FoxP3^+^ regulatory T (Treg) cells and CD11b^+^Gr1^+^ myeloid-derived suppressor cells (MDSCs) that actively drive CD8^+^ T cell exhaustion. Conversely, prostate cancer bone metastases typically display a predominantly osteoblastic or hybrid phenotype. In these lesions, the stroma is heavily dominated by dysregulated bone-forming osteoblasts and cancer-associated fibroblasts (CAFs) that secrete excessive levels of CXCL12 and SCUBE2, orchestrating an immune-cold microenvironment characterized by the absolute physical exclusion of effector T cells and the direct metabolic suppression of NK cells via heightened LAIR1 signaling. Furthermore, this immune landscape undergoes dynamic temporal evolution: the early phase of single-cell metastatic colonization is marked by acute, localized tumor-osteoclast coupling and an influx of tolerogenic dendritic cells, whereas late-stage macro-metastases exhibit extensive extracellular matrix (ECM) fibrosis, severe localized hypoxia, and terminal exhaustion of CD8^+^ T cells (Tex). Categorizing these structured biological profiles is vital for precisely matching nanotechnology-based platforms with the distinct immune-metabolic signatures of individual patients.

### 2.3 Stromal cells

#### 2.3.1 Osteoblasts

Osteoblasts are not only essential for bone matrix synthesis and mineralization but also act as “immune regulatory hubs” within the bone metastasis microenvironment. Tumor cells stimulate osteoblasts to upregulate RANKL expression while suppressing OPG by releasing factors such as PTHrP and IL-6, thereby activating the RANK/NF-κB and MAPK pathways, which drive osteoclast differentiation and bone resorption (89–91). At the same time, osteoblasts secrete CXCL12 and VEGF to recruit circulating tumor cells, promoting their colonization and activating PI3K/Akt and Wnt/β-catenin pathways via integrin signaling, thus enhancing tumor survival and invasiveness (88, 92). Furthermore, osteoblasts have been reported to release IL-6 (and potentially MCP-1) and, in certain contexts, express PD-L1, which contributes to Teff suppression, Tex differentiation, Treg expansion, and the inhibition of CD8^+^ T-cell and NK cell activity in bone metastases (88, 93). Recent evidence also demonstrates that radiotherapy impairs osteoblast function by reducing bone formation, promoting resorption, and creating a microenvironment conducive to tumor persistence and therapy resistance. This occurs through direct damage to osteoblasts, suppression of their proliferation and differentiation, disruption of bone vasculature and matrix, and dysregulation of metabolic signaling pathways such as the RANKL/OPG and Wnt/DKK1 ratios (94). Collectively, osteoblasts serve multifaceted roles in bone metastases: they regulate bone metabolism by modulating the RANKL/OPG balance, facilitate tumor colonization and survival through chemokine and growth factor secretion, contribute to immunosuppressive niche formation, and indirectly promote tumor progression when compromised by radiotherapy. Consequently, targeting osteoblast-mediated mechanisms offers a promising avenue for developing novel therapeutic strategies against bone metastases.

#### 2.3.2 Osteoclasts

Osteoclasts are the primary effector cells responsible for bone resorption, contributing not only to bone destruction but also to the establishment of an immunosuppressive microenvironment through multiple mechanisms during bone metastasis progression. A pivotal study by Lin et al. demonstrated that tumor cells activate tumor-associated osteoclasts by releasing migrasomes enriched with tumor-derived RNA, resulting in the spatiotemporal coupling of “tumor-osteoclast” interactions (34). Once activated, osteoclasts degrade the bone matrix via MMPs and cathepsin K, releasing growth factors that fuel tumor proliferation; additionally, the acidic microenvironment created by bone resorption enhances tumor HIF-1α signaling, thereby promoting drug resistance (40, 95). Beyond their bone-resorptive role, osteoclasts secrete immunosuppressive mediators such as TGF-β and OPN, driving T-cell differentiation into suppressive phenotypes including Tregs and Tex. They also upregulate IDO1 to deplete tryptophan and activate AhR signaling, while simultaneously recruiting MDSCs through CCL3/CCL5, collectively impairing CD8^+^ T-cell and NK cell activity and amplifying immune evasion (32, 57, 87, 89). In summary, osteoclasts serve a dual function within the bone metastatic niche: they mediate bone destruction by degrading the matrix and generating acidic, growth factor-rich conditions that promote tumor progression and drug resistance, while also orchestrating immunosuppression by inhibiting effector immune cells and facilitating immune escape. Consequently, targeting osteoclasts has emerged as a critical therapeutic strategy for the treatment of bone metastases.

#### 2.3.3 Cancer-associated fibroblasts (CAFs)

CAFs are key stromal components of the bone metastasis immune microenvironment, characterized by high heterogeneity and the ability to drive metastatic progression through diverse mechanisms. Within bone metastatic lesions, CAFs secrete abundant ECM remodeling molecules, including chitinase 3-like protein 1 (CHI3L1) and the matrix metalloproteinase MMP2, which restructure the local matrix architecture, promote tumor invasion, and exacerbate osteolytic responses (96, 97). Specific CAF subpopulations, such as PDGFRα^+^ITGA11^+^ CAFs, interact with lymphatic endothelial cell E-selectin (SELE) via ITGA11, thereby activating the SRC-p-VEGFR3-MAPK signaling cascade, which enhances neolymphangiogenesis, increases vascular permeability, and facilitates tumor cell colonization in bone (98, 99). CAFs also indirectly promote osteoclast differentiation by stimulating the RANKL pathway through the secretion of cytokines such as TGF-β and IL-6 (100). At the immunoregulatory level, CAFs recruit MDSCs and Treg cells, leading to suppression of CD8^+^ T-cell and NK cell activity (101, 102). Importantly, CAF functions vary across tumor types: in prostate cancer, they predominantly promote osteoblastic metastasis, whereas in breast cancer, they enhance osteolytic destruction (103, 104). Thus, selectively targeting CAF subsets and their secretory factors represents a promising therapeutic approach for managing bone metastases.

#### 2.3.4 Mesenchymal stem cells (MSCs)

MSCs, a diverse and phenotypically heterogeneous population of stromal cells, exert dual functions within the immune microenvironment of bone metastases. On one hand, they contribute to osteogenic differentiation and structural remodeling; on the other, they support tumor progression through complex cytokine networks. MSCs facilitate tumor cell colonization in bone by secreting proinflammatory mediators such as TGF-β and IL-6 and by regulating ECM components. For instance, breast cancer cells can stimulate bone marrow-derived MSCs to release RANKL, thereby activating osteoclasts and promoting bone resorption (105). In addition, MSCs secrete Sonic Hedgehog (SHH) via the SCUBE2-mediated Hedgehog pathway, inducing osteoblast differentiation and collagen deposition (106, 107). However, this same pathway suppresses NK cell activity through LAIR1 signaling, creating a tumor-supportive immunosuppressive niche. At the immune regulatory level, MSCs secrete CCL2 to recruit MDSCs, which deplete arginine via ARG1 and consequently impair T-cell activity (108, 109). Furthermore, MSCs drive macrophage polarization toward the M2 phenotype, leading to the release of IL-10 and TGF-β, which further dampen antitumor immunity (110, 111). Based on these mechanisms, multiple intervention strategies have been proposed, including targeting SCUBE2 or inhibiting TGF-β signaling, both of which hold promise for reversing the pro-metastatic roles of MSCs. In summary, MSCs are pivotal regulators in the bone metastatic niche and represent a key focus for the development of therapies targeting tumor-stroma interactions.

In summary, unlike primary tumors, bone metastases display the dual hallmarks of dysregulated bone metabolism and profound immunosuppression. These conditions are characterized by distinct alterations in immune cell infiltration patterns, functional states, and microenvironmental signaling pathway activities, collectively contributing to the limited efficacy of current clinical therapies for bone metastases. Nanomaterial-based delivery systems offer a promising solution by enabling the precise and controlled transport of small molecules or gene regulatory factors to metastatic sites. Through the targeted modulation of differential signaling pathways, such approaches can disrupt pro-metastatic and immunosuppressive circuits while restoring the capacity of immune cells to differentiate and polarize effectively within the bone metastatic niche. Thus, nanomaterials specifically designed to target bone metastases hold considerable potential to overcome therapeutic resistance and provide new opportunities for improving clinical outcomes.

## 3 Nanomaterials used for bone metastasis treatment

A pivotal conceptual bridge in optimizing nanomedicine design involves recognizing that nanoplatforms primarily engineered to target structural bone components or tumor cells frequently precipitate profound, secondary immunomodulatory cascades that structurally remodel the tumor immune microenvironment (TIME). For instance, alendronate- or zoledronate-functionalized nanoparticles are explicitly tailored to chelate with hydroxyapatite-rich mineralized matrices to inhibit hyperactive osteoclasts. However, by effectively suppressing osteoclast-mediated bone resorption, these platforms indirectly halt the massive release of matrix-bound transforming growth factor-beta (TGF-β) and osteopontin (OPN) into the niche. As detailed in Section 2.3.2, these osteoclast-derived factors serve as primary instigators of T-cell terminal exhaustion and regulatory T (Treg) cell polarization. Consequently, the secondary consequence of osteoclast inhibition is a sharp localized decline in active TGF-β concentrations, which effectively relieves the transcriptional repression on CD8^+^ cytotoxic T cells and natural killer (NK) cells, converting an immunologically “cold” bone niche into an active, “hot” environment. Similarly, nanoplatforms triggering immunogenic cell death (ICD) via local chemotherapy or sonodynamic therapies do not merely ablate tumor mass; the resulting spatiotemporal release of damage-associated molecular patterns (DAMPs) serves as a potent stimulus that reverses the tolerogenic phenotype of CD11b^+^ dendritic cells (DCs) outlined in Section 2.2.3. This promotes mature DC-mediated antigen presentation and upregulates costimulatory molecules (CD80/CD86), thereby systematically reconnecting structural bone remodeling with long-term antitumor adaptive immunity.

### 3.1 Polymeric nanoparticles (PNPs)

PNPs are colloidal macromolecules ranging in size from 10 to 1,000 nm (112, 113). They serve as versatile carriers in cancer therapy due to their controlled drug release, biocompatibility, and prolonged circulation time (114–116) (Table 2). The composition of PNPs has evolved significantly over time. Initially, nonbiodegradable polymers such as polyethylene glycol (PEG), polymethyl methacrylate (PMMA), polyacrylamide, polystyrene, and polyacrylates were commonly employed (122–124). However, nanoparticles derived from these polymers required rapid clearance to prevent toxicity and chronic inflammation. To overcome these limitations, biodegradable polymers were developed to enhance safety, improve drug release kinetics, and increase compatibility. These include synthetic polymers such as polylactic acid (PLA), poly(lactic-co-glycolic acid) (PLGA), poly(amino acids), and poly(ε-caprolactone) (PCL), as well as natural polymers such as chitosan, alginate, gelatin, and albumin (123, 125, 126). Compared with their predecessors, biodegradable PNPs offer unique advantages including tunable properties, high biocompatibility, and responsiveness to environmental stimuli. Importantly, their surfaces can be functionalized, for example, with bisphosphonates, to specifically target the bone marrow niche and increase drug accumulation at metastatic sites (117).

**TABLE 2:** Polymeric nanoparticles (PNPs).

Modification	Payload	Therapies involved	Level of evidence & model validation	Outcome	Translational bottlenecks & safety profile	References
Alendronate-functionalized porous nanocrystalsomes	Doxorubicin (DOX)	Bone-targeted chemotherapy	*In vitro* characterization + In vivo 4T1 tibial bone metastasis mouse model	Bone-targeted delivery, ↓osteolysis, ↓tumor proliferation, improved local drug retention	Lack of large-animal validation; limited long-term PK/toxicity data; manufacturing complexity	(117)
Transformable supramolecular PNPs (NLG919@CF: Ce6-CD + Fc-pep-PEG)	IDO-1 inhibitor (NLG919)	PDT + immune checkpoint blockade (anti–PD-1)	*In vitro* immune assays + In vivo murine breast cancer bone metastasis model	↓Tregs, ↑CD8^+^ T-cell activity, enhanced anti–PD-1 response, reduced tumor burden and osteolysis	Laser penetration limitations; immune-related toxicity risk; complex supramolecular manufacturing	(25)
PEG-b-PPS polymeric nanocarriers	Gli inhibitor (GANT58)	Hedgehog signaling inhibition	*In vitro* studies + Intratibial/intracardiac breast cancer bone metastasis mouse models + PK/biodistribution assessment	↓Gli signaling, ↓osteoclastogenesis, ↓tumor-induced bone resorption	Potential pathway-specific resistance; lack of clinical validation; long-term accumulation unknown	(118)
Disulfide-crosslinked PEG-PDMAEMA PNPs	TGF-β1 and FOXM1 siRNAs	Gene silencing therapy	*In vitro* TNBC/MG-63 co-culture only	↓EMT, ↓migration and invasion, potential immunomodulatory benefits	No *in vivo* validation; unknown biodistribution; possible cationic-polymer toxicity; delivery efficiency uncertain	(119)
PLGA nanoparticles embedded in thermosensitive PLA hydrogel	Celecoxib (COX-2 inhibitor)	Local delivery postbiopsy	*In vivo* biopsy-induced metastasis murine model	↓M2-like macrophage infiltration, ↓angiogenesis, ↓EMT, prevention of biopsy-induced metastasis	Limited applicability to established metastases; local inflammatory response and degradation kinetics require evaluation	(120)
PEGylated PNPs	STING agonist	Systemic immune activation	*In vivo* 4T1 tibial bone metastasis mouse model	↑CD4^+^/CD8^+^ T-cell activation, ↑IFN-I, ↓osteolysis, ↑Tregs and PD-L1 (transient effect)	Risk of systemic immune overactivation; transient PD-L1/Treg rebound; long-term immune safety unclear	(121)

PEG, polyethylene glycol; PLA, polylactic acid; PLGA, poly(lactic-co-glycolic acid); PNPs, polymeric nanoparticles; IDO-1, indoleamine 2,3-dioxygenase-1; PDT, photodynamic therapy; EMT, epithelial–mesenchymal transition; FOXM1, forkhead box protein M1.

Nanoparticle-based delivery of immunomodulators has shown particular promise in reprogramming the immunosuppressive bone metastatic microenvironment (120, 127). QIN et al. developed transformable NLG919@CF supramolecular nanoparticles by encapsulating the IDO-1 inhibitor NLG919 using Ce6-CD (β-cyclodextrin-conjugated chlorin e6) and Fc-pep-PEG (ferrocene-peptide-PEG) (25). Upon light activation, ROS production oxidizes ferrocene, inducing a morphological shift from spherical nanoparticles to nanofibers, which prolongs retention within the tumor-bone niche. Concurrently, NLG919 is released, inhibiting IDO-1 activity and reversing local immunosuppression by reducing Treg infiltration and restoring CD8^+^ CTL function. In murine models of breast cancer bone metastasis, combining NLG919@CF with photodynamic therapy (PDT) not only suppressed tumor progression and osteolysis but also synergized with anti-PD-1 checkpoint blockade to further potentiate antitumor immunity. This dual-functional nanoplatform highlights how stimulus-responsive PNP architectures can be designed for spatiotemporally controlled drug delivery while simultaneously reprogramming the immune landscape of bone metastases. Nonetheless, challenges remain, including the limited penetration of light into deep bone tissues and uncertainties regarding the durability of T-cell-mediated immunity. Overall, these findings demonstrate that PNPs hold substantial potential for alleviating immunosuppression in bone metastases and restoring effective antitumor immune responses.

In addition to modulating immune cells, PNPs can also target the bone-remodeling axis to both protect bone integrity and suppress tumor growth. Shukla RP et al. developed a functionalized porous nanocrystal system modified with alendronate (alendronate bisphosphonate (ALN) + OA@NCs) (117) for the bone-targeted delivery of oleanolic acid (OA) and ALN to metastatic bone lesions in breast cancer. The porous crystalline framework, fabricated via nanoemulsion crystallization, enables uniform particle size, favorable ζ-potential, and high dual-drug loading efficiency. Compared with OA@NCs alone, ALN + OA@NCs exhibit superior efficacy by integrating two therapeutic functions: (i) direct cytotoxicity against tumor cells through OA-induced ROS generation and apoptosis, and (ii) potent inhibition of RANKL-driven osteoclastogenesis, which reduces osteolysis and slows tumor progression in a 4T1 tibial metastasis model. Key advantages of this system over free drug administration include enhanced bone affinity from ALN functionalization, synergistic dual-action therapy, and improved pharmacokinetics featuring prolonged circulation and sustained drug release at osteolytic sites.

Similarly, Vanderburgh JP et al. engineered a ROS-responsive polymer micelle encapsulating the Gli2 inhibitor GANT58. This design addresses the poor solubility, rapid clearance, and limited tumor penetration of free GANT58 by entrapping the hydrophobic drug within an amphiphilic copolymer, thereby improving systemic delivery and biodistribution. Alendronate conjugation further provides active bone-targeting capability through bisphosphonate-hydroxyapatite affinity, while concurrently delivering localized antiresorptive effects. In preclinical breast cancer bone metastasis models, this system reduced osteolytic lesion area by ∼50% and nearly doubled trabecular bone volume, without adversely affecting osteoblasts or MSCs (118). In summary, alendronate-functionalized PNPs represent a promising bone-targeting strategy. By co-delivering therapeutic agents, they can simultaneously inhibit hyperactive osteoclasts and induce tumor cell death within the bone metastatic microenvironment, thereby suppressing tumor progression and mitigating skeletal destruction.

At the tumor-cell level, PNPs serve as efficient carriers for gene-silencing agents. For instance, Wang et al. developed a stimulus-responsive polymeric delivery platform by co-loading TGF-β1 siRNA and FOXM1 siRNA into disulfide crosslinked PEG-PDMAEMA nanoparticles (119). This nanocarrier was specifically engineered to suppress triple-negative breast cancer (TNBC) cells and their bone metastatic activity. With a self-assembled diameter of approximately 122 nm, the nanoparticles safeguard siRNAs against enzymatic degradation and enable redox- and pH-responsive intracellular release, thereby ensuring silencing efficiency comparable to that of Lipofectamine 2000 in MDA-MB-231 cells. The simultaneous knockdown of TGF-β1 and FOXM1 produced a synergistic antitumor effect, markedly reducing cell viability, migration, invasion, and the adhesion and chemotaxis of metastatic TNBC cells toward MG-63 osteoblast-like cells, processes characteristic of epithelial-mesenchymal transition (EMT) and bone colonization. Compared with conventional siRNA vectors, this platform offers several advantages: (i) stimuli-triggered release reduces off-target effects while enhancing siRNA bioavailability within the reductive tumor microenvironment; (ii) dual-gene silencing effectively counteracts EMT by downregulating vimentin and upregulating E-cadherin, producing stronger inhibition than single-siRNA strategies; and (iii) high stability and transfection efficiency, coupled with serum resistance and long-term storage capacity, increase reproducibility and translational promise. Nonetheless, the immediate clinical relevance remains constrained by notable gaps: current findings are restricted to *in vitro* studies without validation in bone metastasis models, biodistribution and pharmacokinetics remain uncharacterized, and delivery efficacy to metastatic bone lesions has not been demonstrated. Furthermore, while EMT endpoints were examined, downstream outcomes such as apoptosis, cell cycle arrest, and long-term clonogenic survival were not investigated, leaving oncocidal potential only partially defined. Overall, PEG-PDMAEMA-based siRNA co-delivery nanoparticles significantly advance precision gene silencing of oncogenic and EMT-associated pathways in TNBC cells, highlighting substantial anti-metastatic promise at the cellular level.

Collectively, these findings highlight the versatility of polymeric nanomedicines in targeting diverse components of the tumor microenvironment, including cancer cells, immune subsets such as T cells, NK cells, macrophages, and MDSCs, as well as bone-resorbing cells. By simultaneously modulating these niches, polymer-based systems can interrupt the pathological feedback loop that drives bone metastasis, achieving targeted and sustained therapeutic delivery. Despite their chemical versatility, the ultimate clinical translation of polymeric NPs is acutely bottlenecked by structural batch-to-batch inconsistencies during scale-up synthesis and the latent long-term systemic toxicity induced by the non-cleavable accumulation of polymeric metabolic byproducts.

### 3.2 Lipid-based nanomaterials

Research on lipid-based nanomaterials has expanded rapidly, with three major classes, liposomes (LNPs), solid lipid nanoparticles (SLNs), and nanostructured lipid carriers (NLCs), receiving substantial attention in both preclinical and clinical investigations (128, 129) (Table 3). Liposomes are spherical vesicles typically composed of unilamellar or multilamellar phospholipid bilayers, ranging in size from approximately 20 nm to over 1 µm (133). Their characteristic architecture, featuring a hydrophilic aqueous core enclosed by a hydrophobic bilayer, allows for the simultaneous encapsulation of hydrophilic and hydrophobic therapeutic agents, depending on drug properties (134). Hydrophilic compounds are generally sequestered within the central aqueous cavity, whereas hydrophobic agents integrate into the lipid bilayer. This encapsulation strategy not only enhances drug stability but also shields encapsulated molecules from degradation during systemic circulation, thereby improving delivery efficiency (135).

**TABLE 3:** Lipid–based nanomaterials.

Modification	Payload	Therapies involved	Level of evidence and model validation	Outcome	Translational bottlenecks and safety profile	References
Tetracycline-modified lipid nanosomes (HC&HP@TNL)	Sodium bicarbonate + sodium dihydrogen phosphate	Behavior-targeted physical killing	Tumor–osteoclast co-culture + In vivo murine bone metastasis model + anti-PD-1 validation	Inhibited tumor–osteoclast coupling, induced ICD via CaP crystal formation, prevented initial metastatic seeding	Long-term biosafety unknown; incomplete PK characterization; tumor heterogeneity validation lacking; manufacturing complexity	(34)
Bone-targeting lipid nanoparticles (BT-LNP) modified with bisphosphonate	siRNA targeting TUBB3	Gene silencing of anoikis resistance	*In vitro* anoikis-resistance assays + In vivo prostate cancer bone metastasis mouse model	↓anoikis resistance, ↓cell invasion and metastasis, suppressed prostate cancer bone colonization	Potential resistance bypass mechanisms; uncertain long-term PK/stability; possible liver/spleen accumulation; limited tumor-spectrum validation	(130)
Solid lipid nanoparticles (SLN) coloaded with doxorubicin + protective agents	Doxorubicin ± bone-protective actives	Chemotherapy + bone retention	*In vivo* chemotherapy-related bone loss rodent model with micro-CT assessment	Protected against bone loss during DOX therapy, reduced osteotoxicity, preserved bone structure	No active bone targeting; unclear efficacy in established metastases; insufficient long-term PK/toxicity evaluation	(131)
A bisphosphonate-modified LNP (LNP)	miRNA (tumor suppressor)	Gene therapy + bisphosphonate targeting	*In vitro* studies + *in vivo* breast cancer bone metastasis mouse model	↓tumor weight, restored trabecular structure, induced cancer apoptosis and osteoclast suppression	Off-target miRNA effects; biodistribution and immune safety unknown; scalability and clinical translation challenges	(132)

LNPs, liposomes; SLNs, solid lipid nanoparticles; NLCs, nanostructured lipid carriers; TNL, tetracycline-modified lipid nanosome; ICD, immunogenic cell death; TUBB3, class III β-tubulin.

Liposomes have more recently been engineered to intervene in the early spatial and temporal crosstalk between tumor cells and bone-resorbing osteoclasts. Gu et al. reported the development of a tetracycline-modified LNPs system (TNL), which leverages the acidic microenvironment generated by tumor-activated osteoclasts (termed tumasteoclasts) to trigger the localized release of phosphate ions, resulting in the *in situ* formation of calcium phosphate crystals (34). These nanoparticles physically disrupt tumor cell membranes and suppress migrasome formation, thereby uncoupling the tumor-osteoclast interaction and halting early metastatic colonization. Unlike conventional molecular-targeted therapies, this strategy emphasizes “behavioral targeting,” focusing on pathological cell-cell interactions rather than isolated signaling pathways. The platform integrates bone-targeting ligands (tetracycline), pH responsiveness, and spatially restricted mineral precipitation to ensure precise localization and minimize systemic toxicity. Furthermore, the induction of immunogenic cell death enhances antitumor immune activation, and when combined with PD-1 blockade, the system produces potent synergistic effects, nearly abolishing bone metastasis in murine models. This dual-action design represents a paradigm shift in bone-targeted nanotherapy, uniting mechanical disruption with immune modulation. Despite compelling preclinical evidence, key translational challenges remain, including long-term biosafety, pharmacokinetic profiling, and validation across diverse tumor types. Nevertheless, by targeting the dynamic tumor-osteoclast niche rather than static molecular markers, this lipid-based nanoplatform demonstrates strong potential to prevent skeletal metastases at their earliest stage.

In parallel with this strategy, bone-targeted LNPs (BP-LNPs) functionalized with bisphosphonates such as alendronic acid or zoledronate have been shown to selectively accumulate in hydroxyapatite-rich bone through high-affinity ligand chelation (130). These demonstrations underscore the remarkable tunability and tissue-specific targeting capacity of liposomal systems, thereby opening new therapeutic avenues for managing bone metastases. Despite these advantages, several hurdles remain, including the need for comprehensive safety evaluations, detailed pharmacokinetic profiling, and validation across diverse tumor models. Taken together, liposomes represent a versatile platform that holds considerable promise for bone metastasis therapy by simultaneously modulating immune responses and bone metabolism.

Compared with conventional liposomal vectors, SLNs represent a second-generation lipid-based delivery platform, characterized by a solid lipid core that promotes sustained release and improved formulation stability. Radicchi et al. encapsulated doxorubicin into SLNs (SLN-Dox) and assessed its effects in a Stage IV breast cancer metastasis murine model (131). Strikingly, SLN-Dox not only preserved the antitumor activity of free doxorubicin but also protected skeletal integrity, maintaining growth plate, cancellous, and compact bone microarchitecture in both tumor-bearing and healthy mice, outcomes unattainable with unencapsulated doxorubicin. Mechanistically, the controlled release profile of the lipid matrix reduced systemic drug peaks, thereby lowering osteoblast apoptosis and TGF-β-mediated osteolysis while mitigating chemotherapy-induced bone degradation even in non-tumor-bearing hosts. Micro-CT and electron microscopy analyses confirmed preserved bone structure in SLN-Dox-treated groups, suggesting direct protection of osteogenic niches from chemotoxic injury. Beyond drug delivery, the SLN platform appears to inherently regulate bone metabolism by reducing osteoclast activation, indirectly enhancing osteoblast resilience. Nonetheless, certain limitations temper translational readiness: the study primarily addressed preventive effects on bone loss in mice without exploring long-term outcomes, pharmacokinetics, or direct efficacy against established bone metastases. Moreover, the absence of active bone-targeting ligands (e.g., bisphosphonate modifications) may lead to broader systemic distribution, reducing the precision achieved by bone-homing lipid systems. Overall, SLN-Dox exemplifies how solid lipid nanocarriers can couple potent chemotherapy with skeletal preservation, offering a promising paradigm for integrating bone health protection into oncologic nanomedicine.

Ionizable LNPs functionalized with bone-homing ligands have recently emerged as a highly promising strategy for overcoming anoikis resistance-driven prostate cancer bone metastasis. Dong et al. designed a bisphosphonate-modified LNP (BT-LNP) encapsulating siRNA against TUBB3, a microtubule β-III tubulin isoform markedly upregulated in anoikis-resistant prostate cancer cells (132). Owing to their strong affinity for hydroxyapatite-rich bone tissue, BT-LNPs preferentially accumulate within metastatic bone niches, where systemic administration enables localized TUBB3 silencing. This knockdown disrupts the αvβ3/FAK/Src signaling cascade, reverses EMT, and suppresses invasion, migration, and detachment-induced apoptosis resistance *in vivo*. Mechanistically, the design integrates skeletal targeting through bisphosphonate ligands with gene-specific silencing by siRNA, thereby disabling integrin-mediated survival pathways in metastatic tumor cells. Consequently, BT-LNP therapy significantly reduces prostate cancer bone colonization and metastatic burden in animal models, highlighting its dual capacity for potent antitumor efficacy and niche-specific precision delivery. This approach exemplifies the adaptability of lipid-based nanomaterials, where rational surface engineering (bisphosphonate conjugation) and tailored payload selection (siTUBB3) synergize to intercept metastasis at the stage of anoikis resistance. Unlike conventional chemotherapy, which relies on broadly cytotoxic mechanisms, this strategy directly addresses the survival adaptations that facilitate metastatic establishment. Nevertheless, several challenges remain: targeting a single gene leaves open the possibility of TUBB3-independent resistance; the long-term stability and pharmacokinetic behavior of bisphosphonate-LNP conjugates require further refinement; and efficacy across heterogeneous prostate cancer genotypes or non-bone metastatic sites has not yet been determined. Despite these limitations, bone-homing BT-LNPs represent a mechanistically innovative and highly specific siRNA delivery platform, advancing the potential of lipid-based nanotherapies to block metastatic prostate cancer by directly modulating anoikis resistance pathways.

Lipid-based nanomaterials, including liposomes, SLNs, and NLCs, have shown broad therapeutic potential in addressing bone metastases by simultaneously targeting tumor progression and bone metabolic imbalance. Together, these platforms represent a multifaceted treatment paradigm that integrates direct tumor inhibition, modulation of the bone microenvironment, and activation of antitumor immunity within a bone-specific delivery system. While lipid-based nanoplatforms possess established regulatory precedents, their reliance on high-affinity bone-targeting ligands (e.g., bisphosphonates) presents a double-edged sword, as chronic systemic exposure carries the risk of disrupting normal skeletal remodeling and inducing jaw osteonecrosis or atypical fractures.

### 3.3 Cell membrane-wrapped biomimetic nanomaterials

Cell membrane-wrapped nanomaterials have emerged as a promising class of biomimetic platforms that combine the natural functional attributes of cell membranes with the engineered performance of nanocarriers, thereby enabling precise tumor targeting, immune modulation, and enhanced pharmacokinetics. To ensure strict biological relevance to skeletal malignancies, top priority must be assigned to platforms validated directly within intraosseous lesions. For instance, Li et al. engineered dual-targeting semiconducting polymer nanocomposites (SPFeNOC) cloaked with hybrid membranes derived from both osteoclasts and tumor cells, enabling simultaneous homotypic and heterotypic recognition within bone metastatic microenvironments (136). These nanocomposites preferentially accumulated in bone lesions and produced strong therapeutic outcomes by integrating sonodynamic and chemodynamic effects, thereby disrupting the tumor-bone feedback loop. In parallel, Liu et al. designed TMTP1-modified extracellular vesicles (EVs) to target PIK3CA-mutant non-small cell lung cancer (NSCLC) bone metastases (137). These EVs effectively suppressed PD-L1 expression and enhanced immune activation by promoting CD8^+^ T-cell infiltration and driving M1 macrophage polarization, ultimately overcoming immune checkpoint resistance within metastatic niches. Similarly, Chen et al. developed red blood cell membrane-coated nanoparticles for the co-delivery of piceatannol and doxorubicin, achieving prolonged systemic circulation and tumor-responsive release (138). This platform effectively inhibited metastatic colorectal cancer by attenuating tumor stemness through suppression of the Hippo/YAP1/SOX9 signaling pathway.

As an extended future perspective rather than an immediate skeletal solution, alternative natural biomimetic approaches targeting distant immune axes warrant mention. Recently, Xu et al. reported an intriguing study where oral garlic-derived nanoparticles (GNPs) mimic cellular membrane features to selectively activate interferon-γ-producing γδT cells within the intestinal mucosa, subsequently migrating to distant visceral tumors (76). However, the therapeutic efficacy of these gut-tumor axis agents has not yet been validated in bone metastatic disease. To successfully translate such non-skeletal platforms to bone lesions, investigators must overcome critical additional physiological obstacles, including the engineering of high-affinity bone-homing ligands (e.g., bisphosphonate conjugation) to facilitate active extravasation across the dense, restrictive blood-bone barrier.

Collectively, these investigations underscore the broad adaptability of membrane-camouflaged nanoplatforms across multiple tumor settings. While Xu et al.’s GNP-based system introduces a novel oral immune-priming strategy, other approaches exploit membrane engineering to enable dual-organ targeting, reverse immune checkpoint resistance, or suppress tumor stemness. Moving forward, critical translational challenges remain to be addressed, including scalable manufacturing, characterization of immune biodistribution within bone metastatic niches, and thorough long-term biosafety assessments across species. Even so, cell membrane-wrapped nanomaterials stand out as a promising frontier for precision cancer immunotherapy and metastasis-specific interventions. Biomimetic membrane-wrapped platforms excel in immune evasion, yet they are significantly constrained by the lack of standardization across donor cell lines, the complexity of clinical-grade sterilization protocols, and persistent host immunogenicity risks from trace non-self surface proteins.

### 3.4 Protein-based nanoparticles

Protein-based nanoparticles are nanoscale carriers composed of natural or engineered proteins, such as zein, albumin, or ferritin, designed to encapsulate and deliver therapeutic agents (139, 140). These platforms offer excellent biocompatibility, biodegradability, and inherent targeting capabilities, making them attractive candidates for cancer therapy and bone-specific drug delivery. A recent study by Ganesan et al. (2024) reported the development of cryoprotective isoliquiritigenin-zein phosphatidylcholine nanoparticles (ISL@ZLH NPs) for oral or systemic administration in a breast cancer bone metastasis model. These protein-lipid hybrid nanoparticles effectively inhibited tumor proliferation and osteoclast-mediated bone resorption by suppressing the JAK-STAT signaling pathway in RAW264.7 precursor cells, thereby reducing TRAP-positive osteoclastogenesis and preserving bone microarchitecture *in vivo*. Notably, treated animals exhibited significantly fewer lytic lesions and maintained bone integrity, with histological analyses confirming that tumor burden remained largely confined within the marrow cavity rather than infiltrating cortical bone (141).

The zein-based nanoparticles integrate multiple advantageous features: the biodegradable protein core provides stable encapsulation of the hydrophobic isoliquiritigenin cargo, while phosphatidylcholine enhances particle dispersibility and cellular uptake. Cryoprotectants further improve physical stability, allowing long-term storage without loss of efficacy and enabling oral administration. Mechanistically, the ISL payload exerts anti-osteoclastogenic effects by inhibiting STAT3 phosphorylation and downstream transcription factors such as NFATc1, thereby disrupting the cancer-induced bone destruction feedback loop. Simultaneously, the nanoparticles impair breast cancer cell migration and proliferation through suppression of PI3K-Akt-mTOR signaling and MMP-mediated pathways, demonstrating dual functionality in tumor suppression and skeletal protection (142).

Despite their promise, several limitations remain. Current evidence is largely confined to murine models of breast cancer bone metastasis, necessitating validation in additional tumor types and larger animal systems. Furthermore, the pharmacokinetic profile of zein nanoparticles and potential off-target effects, including immunogenicity and systemic biodistribution, require thorough investigation. Nevertheless, by integrating a protein-based nanocage architecture with bioactive flavonoids that target the JAK-STAT signaling pathway, the ISL@ZLH NP platform represents an innovative nanoformulation capable of dual-action therapeutic intervention, simultaneously preserving bone integrity and suppressing metastatic tumor growth within skeletal niches. Living cell-based modulators offer unparalleled active homing capabilities; however, ensuring the biosafety of genetic modifications, preventing uncontrolled lineage differentiation within the tumor niche, and mitigating inherent tumorigenicity risks remain major translational barriers.

### 3.5 Living cell-based biomimetic materials

Living cell-based biomimetic materials have recently emerged as transformative platforms for modulating the bone metastatic microenvironment and enhancing immunotherapeutic responses. Wang et al. engineered programmed PD-1-expressing hematopoietic stem cells (HSCs-PD-1) to deliver the TGF-β inhibitor SB-505124 directly into the bone marrow of metastatic tumor-bearing mice (143). These “living drugs” exploit the natural homing capacity of stem cells to selectively return to bone tissue, self-renew *in situ*, continuously express PD-1, and release SB-505124 to competitively block TGF-β signaling in CD4^+^ T cells. Consequently, this approach reprograms the local immune milieu by promoting TH1/TH2 differentiation, restoring CD8^+^ T-cell activity, and mitigating PD-L1-mediated immunosuppression, resulting in superior antitumor efficacy compared with systemic anti-PD-L1 antibody therapy in bone metastasis models.

Complementing this strategy, Liu et al. developed a genetically engineered Lactococcus lactis strain as a live probiotic vector capable of localized immunotherapy (144). Although primarily studied in pleural or systemic metastasis models, engineered L. lactis strains expressing immunostimulatory cytokines or tumor antigens have been shown to efficiently activate DCs, CD8^+^ T cells, NK cells, and macrophages within metastatic niches following intrapleural or nodal administration. Their intrinsic ability to colonize hypoxic microenvironments and recruit antigen-presenting cells suggests that direct delivery into the bone marrow or peritumoral bone niches may represent a viable strategy for the treatment of bone metastases.

Collectively, these cell-based biomimetic platforms, engineered HSCs delivering TGF-β blockade and probiotic bacteria designed for immune activation, provide two complementary strategies: one reshapes the immunosuppressive bone marrow niche from within, while the other supplies external immunostimulatory signals to enhance antigen presentation and effector cell infiltration. Although translation to bone metastatic disease will require careful validation of targeted delivery and long-term safety, these living biomaterials exemplify a new paradigm in which dynamic, self-renewing therapeutics can overcome the distinctive immune challenges of the bone metastatic microenvironment.

### 3.6 Lipid-polymer hybrid nanoparticles

Lipid-polymer hybrid nanoparticles, typically comprising a biodegradable polymeric core (e.g., PLGA or zein) surrounded by a phospholipid or lipid-PEG shell, integrate the mechanical stability and controlled release properties of polymers with the biocompatibility and membrane-fusion capabilities of lipids. Zheng et al. engineered an amphiphilic hybrid by incorporating pyrazinoquinoxaline-based AIEgens into a PLGA core coated with a lipid-PEG shell, producing near-infrared (NIR)-II-emissive 4TPQ nanoparticles for deep-tissue phototheranostic intervention in breast cancer bone metastases (145). This system achieves high quantum yield and tissue penetration for fluorescence imaging, while NIR-II irradiation simultaneously induces photothermal and photodynamic effects, ablating metastatic lesions with minimal off-target toxicity, demonstrating a combined diagnostic and therapeutic capability in a single bone-targeted formulation.

Complementing this strategy, polymer-lipid hybrids have also been leveraged to modulate antitumor immunity within the bone marrow microenvironment. Guo et al. encapsulated the STING agonist cGAMP in a PLGA-lipid hybrid nanoparticle, achieving sustained release in hypoxic bone lesions, where early cytokine induction and enhanced T-cell priming reversed local immunosuppression and attenuated osteolysis in murine models of bone metastasis (146). In a similar vein, immunoliposome-polymer hybrids have been engineered to deliver CRISPR/Cas9 gRNA complexes targeting IL30 in prostate cancer cells: these Cas9hIL30 PSCA nanoparticles integrate a biodegradable polymeric core with a lipid bilayer functionalized by anti-PSCA antibodies, enabling precise gene editing within metastatic niches, reducing tumor burden, and prolonging survival (147).

Collectively, these studies demonstrate that lipid-polymer hybrid nanosystems combine the structural stability and controlled-release capabilities of polymeric cores with the biocompatibility and surface-functionalization versatility of lipid shells, enabling dual-modal imaging and therapy, immune microenvironment modulation, and precise gene editing within bone metastatic niches. Future efforts should focus on optimizing bone-homing ligands, conducting comprehensive long-term safety assessments, and establishing scalable manufacturing processes to facilitate the clinical translation of these multifunctional platforms for the treatment of skeletal metastases.

### 3.7 Organic-inorganic hybrid nanomaterials

Organic-inorganic hybrid nanomaterials are composite nanosystems that combine organic constituents (such as polymers, lipids, or biomolecules) with inorganic components (including metals, metal oxides, or mineral salts), merging the biocompatibility and functional versatility of organics with the structural stability and physicochemical reactivity of inorganics. These hybrid platforms are engineered to support multifunctional therapeutic strategies, encompassing drug delivery, phototherapy, immunomodulation, and bone regeneration (Table 4). Shen et al. developed a hierarchical organic-inorganic hybrid nanoparticle system to address the complex challenges of breast cancer bone metastasis (127). This platform integrates liquid metal (LM) nanoparticles with a zeolitic imidazolate framework (ZIF-8) and mesoporous silica, establishing a responsive cascade for sequential drug release. The outer ZIF-8 shell first delivers curcumin to suppress tumor-associated stromal remodeling, including TGF-β signaling, thereby enhancing vascular permeability and immune cell infiltration. Upon NIR irradiation, the LM core generates mild photothermal heating, disrupting the silica layer and releasing doxorubicin to amplify local cytotoxicity. This combinatorial approach illustrates the unique capacity of organic-inorganic hybrid nanomaterials to coordinate tumor microenvironment modulation with spatiotemporally controlled drug delivery. By remodeling the stromal niche and synchronizing photothermal and chemotherapeutic effects, the system achieves enhanced tumor suppression while preserving bone integrity, highlighting the therapeutic versatility of hybrid nanostructures that bridge chemical sophistication with clinical applicability.

**TABLE 4:** Organic–inorganic hybrid nanomaterials.

Modification	Payload	Therapies involved	Level of evidence and model validation	Outcome	Translational bottlenecks and safety profile	References
Mesoporous Si-coated liquid metal @ ZIF-8 with bone seeking ligand	Curcumin + Doxorubicin	Mild photothermal therapy + chemotherapy	*In vitro* stromal-remodeling studies + *in vivo* 4T1 bone metastasis mouse model + NIR-triggered therapy validation	Remodeling tumor stroma: ↓TGF-β secretion, degraded ECM, ↑CD4^+^/CD8^+^ T-cell infiltration, inhibited tumor–osteoclast crosstalk and osteolysis	Uncertain liquid-metal biodegradation; liver/spleen accumulation risk; limited NIR penetration; manufacturing complexity	(127)
Semiconducting polymer and Fe_3_O_4_ coloaded in ^1^O_2_-responsive carrier (ASPN(FP))	SPN + Fe_3_O_4_	Sono ferroptosis (sonodynamic therapy + Fenton)	*In vitro* ferroptosis/ROS studies + *in vivo* 4T1 bone metastasis mouse model	Amplified oxidative damage (^1^O_2_, ·OH), induced ferroptosis, suppressed tumor growth, limited metastasis, possible ICD release for immune activation	Long-term Fe_3_O_4_ accumulation unknown; potential bone marrow oxidative toxicity; ultrasound standardization challenges	(148)
Alendronate modified PEGylated lipid–polymer nanoparticle with BBT FT DA donor	Bortezomib + Fe^2+^	NIR II photothermal + chemotherapy + CDT	*In vitro* responsive-release studies + *in vivo* 4T1 bone metastasis mouse model	Acid/H_2_O_2_-triggered release, synergistic PTT/CDT/chemo, inhibited tumor growth and osteolysis, bone repair	Fe-mediated oxidative toxicity risk; uncertain NIR-II safety; unclear long-term clearance and biodistribution	(149)
Graphene modified CePO_4_ nanorods in chitosan scaffold	CePO_4_ + GO in scaffold	Photothermal + osteo-regenerative scaffold	*In vivo* postoperative bone metastasis mouse model with osteogenesis evaluation	Regulated macrophage polarization (favor M1), improved osteo-induction, tumor suppression, bone regeneration	Potential graphene-induced inflammation; CePO_4_ retention concerns; scaffold biodegradation and implantation limitations	(150)
Calcium phosphate–polymer hybrid micelles	Zoledronate + Docetaxel	Chemotherapy + osteoclast inhibition	*In vitro* osteoclast inhibition + *in vivo* PC-3 bone metastasis model	Bone-targeting release, suppressed osteoclastogenesis, reduced tumor proliferation and bone lesions	Repeated bisphosphonate exposure toxicity; lack of large-animal validation; uncertain long-term bone retention	(151)
CuP@PPy ZOL NPs (CuP core + polypyrrole shell)	Fenton catalyst (Cu) + ZOL	NIR II photothermal + chemodynamic therapy	*In vitro* bone-remodeling studies + *in vivo* 4T1 bone metastasis mouse model	Dual PTT/CDT, inhibited osteoclast differentiation, promoted osteoblasts, remodeled bone microenvironment, reversed osteolysis	Copper accumulation and hepatotoxicity concerns; dependence on endogenous H_2_O_2_; long-term biosafety undefined	(152)

ZIF-8, zeolitic imidazolate framework-8; SPN, semiconducting polymer nanoparticle; CDT, chemodynamic therapy; PTT, photothermal therapy; NIR-II, near-infrared region II; ZOL, zoledronate; ICD, immunogenic cell death.

Beyond the hierarchically programmed LM@ZIF-8@SiO_2_ nanoparticles, a growing array of organic-inorganic hybrid platforms has demonstrated the potential of integrating stimuli-responsive release, multimodal therapy, and bone-tropic targeting to overcome the complex challenges of skeletal metastases. For instance, semiconducting polymer-ferrite composites can be ultrasound-activated to enhance reactive oxygen species production and induce ferroptotic tumor cell death, whereas NIR-II emissive polymer-metal hybrids provide deep-tissue fluorescence imaging alongside synergistic photothermal, chemodynamic, and chemotherapeutic interventions (148, 149). Similarly, calcium phosphate-polymer hybrids co-deliver bisphosphonates and taxanes to suppress osteoclast-mediated bone resorption while exerting direct antitumor cytotoxicity (150). Graphene-CePO_4_ scaffolds further combine photothermal ablation with M2 macrophage polarization to promote osteogenesis (151). Ligand- or antibody-functionalized nanosystems, such as bisphosphonate-decorated LM particles or CD71-targeted calcium phosphosilicate carriers, achieve preferential accumulation in bone lesions and selective payload delivery to metastatic cancer cells (152).

In summary, organic-inorganic hybrid nanomaterials exemplify a versatile design paradigm for bone metastasis theranostics, uniting the structural tunability and drug-loading capacity of organic matrices with the functional stability and stimuli responsiveness of inorganic components. These systems enable sequential, microenvironment-triggered payload release, multimodal therapeutic strategies, and precise bone targeting via bisphosphonate or antibody ligands. They simultaneously remodel the tumor stroma, inhibit osteoclast-driven bone resorption, activate antitumor immunity, and promote osteogenesis. Although preclinical studies have demonstrated significant improvements in tumor suppression, immune infiltration, and bone preservation, future work must focus on optimizing skeletal homing specificity, evaluating the long-term biosafety and biodegradation of inorganic elements, and establishing scalable manufacturing processes to accelerate the clinical translation of these multifunctional hybrid platforms.

### 3.8 Comparative analysis and critical synthesis of emerging nanoplatforms

To advance from a descriptive overview to a critical evaluation, it is essential to systematically balance the relative merits and translational constraints of the primary nanoplatform categories investigated for bone metastasis. Polymeric nanoparticles (PNPs) offer exceptional chemical flexibility and highly tunable drug release kinetics, making them uniquely suited for multi-payload co-delivery. However, their clinical translation is frequently hindered by challenges in scaling up reproducible, batch-to-batch polymer syntheses and the risk of chronic tissue inflammation driven by non-cleavable degradation byproducts. Lipid-based nanoplatforms (including liposomes, SLNs, and ionizable LNPs) exhibit superior biocompatibility and a more direct path to clinical scaling due to established regulatory precedents. Nonetheless, their translational readiness for skeletal lesions is often limited by physical instability under physiological shear stress, lower loading capacity for highly hydrophobic agents, and a pronounced tendency for off-target sequestration in the liver and spleen rather than the mineralized bone matrix unless heavily surface-functionalized. Cell membrane-wrapped biomimetic nanomaterials excel in immune evasion and exploit native homing axes (e.g., tumor-homing or bone marrow-homing affinities), thereby maximizing target site delivery. Yet, their fatal bottlenecks reside in the complex, low-yield extraction processes of donor membranes, strict requirements for clinical-grade sterilization, and latent risks of host immunogenicity. Finally, organic-inorganic hybrid systems integrate the structural stability and precise stimuli-responsiveness (e.g., pH, ROS, or NIR-II sensitivity) of inorganic cores with the versatile encapsulation efficiency of organic shells. While they demonstrate unmatched multi-modal therapeutic efficacy in preclinical setups, their persistent long-term tissue accumulation, potential heavy metal toxicities (e.g., from Fenton-catalytic copper or iron entities), and unmapped long-term degradation kinetics represent substantial clinical validation barriers. A rigorous, multi-dimensional comparison of these platforms is systematically summarized in the updated (Tables 2, 3).

## 4 Current research gaps and limitations

Despite considerable advances in bone metastasis therapeutics, many scientific and clinical challenges remain, constraining the widespread application and limiting further improvements in treatment efficacy.

### 4.1 Limitations of preclinical models

Existing animal models, particularly murine systems, are limited in their ability to fully recapitulate the chronic progression and heterogeneity of human bone metastases. Key features, such as the dynamic evolution of the immune microenvironment and long-term interactions between osteoclasts and tumor cells, are inadequately represented in short-term experimental models. Moreover, current platforms often fail to mimic multifocal bone metastases or the synergistic impact of concurrent extrabone metastatic sites, restricting the systemic evaluation of therapeutic mechanisms. The inherent complexity of the bone microenvironment, including hypoxia, hypercalcemia, and metabolic reprogramming, further complicates accurate *in vitro* modeling, thereby hindering precise assessment of nanomaterial targeting efficiency and immunomodulatory effects.

### 4.2 Unresolved mechanisms of immune resistance

The mechanisms underlying the resistance of bone metastases to immune checkpoint blockades (ICBs)remain incompletely understood. Comprehensive studies examining the dynamic balance of immunosuppressive cells, such as Tregs and MDSCs, within the metastatic niche, and their interactions with osteoclast activation, are still lacking, limiting the development and optimization of targeted therapeutic strategies.

### 4.3 Deepening the physiological and pharmacological barriers to clinical translation

To properly contextualize the clinical applicability of bone-targeted nanomedicines, several formidable physiological and pharmacological hurdles must be critically expanded. First, the actual delivery efficiency of systemic nanoplatforms to active bone metastatic sites remains exceptionally low—typically under 1%–2% of the injected dose. This is heavily restricted by the dense mineralized cortical bone architecture and the protective blood-bone barrier, which severely limits vascular extravasation into the bone marrow niche. To compensate, investigator-designed platforms heavily rely on high-affinity bone-homing ligands (e.g., bisphosphonates or tetracyclines); however, this structural modification introduces unexpected biodistribution risks, causing off-target accumulation in healthy, non-compromised skeletal tissues and potentially disrupting physiological, homeostatic bone remodeling. Second, the long-term biosafety, systemic clearance pathways, and degradation kinetics of many synthetic polymers and inorganic matrices remain uncharacterized. Prolonged circulation frequently results in massive entrapment within the mononuclear phagocyte system (MPS)—primarily localized in the liver and spleen—instigating chronic hepatotoxicity, splenotoxicity, or transient immunogenicity. For multi-modal organic-inorganic systems employing transition metals (such as Fenton-catalytic copper, manganese, or iron components), the lack of definitive renal or hepatic clearance mechanisms over extended periods raises significant safety red flags regarding cumulative oxidative stress and tissue necrosis. To bridge the widening chasm between preclinical success and clinical implementation, future nanomedicine research must prioritize rigorous pharmacokinetic modeling in large animals, long-term (>6 months) longitudinal biosafety screening, and the establishment of scalable, Good Manufacturing Practice (GMP)-compliant chemical synthesis protocols.

### 4.4 Lack of clinical validation

Despite the advanced development of materials science, a formidable chasm remains between laboratory success and clinical implementation. Most emerging therapies, including bioinspired nanodelivery systems and gene-edited “living drugs,” remain strictly at the preclinical stage and lack robust evidence from multicenter, large-sample clinical trials. For instance, while the anti-RANKL antibody denosumab has been proven to significantly prolong the time to skeletal-related events (SREs) in phase III studies, its synergistic survival benefit when combined with immune checkpoint blockade (ICB) remains clinically unproven within the immunosuppressive bone niche. Furthermore, current clinical investigations predominantly focus on patients with solitary bone metastases, providing limited systematic evaluation of therapeutic strategies for individuals with multifocal lesions or concurrent extra-bone visceral metastases, such as those affecting the liver or lungs. Consequently, bridging this translational gap requires a paradigm shift in trial design to better evaluate targeted nanomedicines in clinically realistic, polymetastatic scenarios.

### 4.5 Category-specific technological solutions to overcome translational bottlenecks

To advance beyond generic critiques, resolving the unique material-specific barriers outlined in Section 3 requires the integration of targeted bioengineering solutions. For lipid platforms, the risk of ligand-induced bone abnormalities can be mitigated through the synthesis of multi-functional ionizable lipids that possess transient, pH-dependent bone affinity, ensuring drug release exclusively within the acidic resorptive pit without prolonged attachment to healthy hydroxyapatite. For biomimetic membrane systems, the bottleneck of donor heterogeneity and immunogenicity can be addressed by implementing high-throughput membrane proteomics screening and microfluidic-based sequential washing, which preserves essential homing receptors while removing highly polymorphic major histocompatibility complex (MHC) fractions. Finally, the biosafety and tumorigenicity concerns surrounding living cell-based vectors can be strictly controlled by integrating inducible suicide gene switches—such as the inducible caspase-9 (iCasp9) or herpes simplex virus thymidine kinase (HSV-tk) circuits—allowing clinicians to clear the cellular vehicles via exogenous small-molecule administration immediately after the therapeutic window closes.

Most emerging therapies, including bioinspired nanodelivery systems and gene-edited “living drugs,” remain at the preclinical stage and lack evidence from multicenter, large-sample clinical trials. For instance, denosumab has been shown to prolong the time to skeletal-related events (SREs) in phase III studies, yet its survival benefit when combined with ICB remains unproven. Current clinical investigations predominantly focus on patients with solitary bone metastases, providing limited systematic evaluation of therapeutic strategies for individuals with multifocal lesions or concurrent extrabone metastases, such as those affecting the liver or lungs).

## 5 Forward-looking strategies and actionable roadmaps for the next decade

To propel the field of bone metastasis nanomedicine from preclinical experimentation to clinical reality over the next 5–10 years, research must pivot away from descriptive screening toward actionable, mechanistically driven paradigms centered on four prospective pillars:

1. Multi-omics-guided precision nanodesign: Future nanoplatform developments should be fundamentally guided by patient-specific tumor landscapes. By utilizing single-cell RNA sequencing (scRNA-seq) and spatial transcriptomics to dissect the precise immune-subcellular architecture of bone lesions across different primary origins, investigators can map localized immune cold/hot topologies. This molecular profiling will enable the customized formulation of nanomodulators tailored to target the exact dominant immunosuppressive cell type or checkpoint signature unique to an individual’s metastatic niche.
2. Temporally programmable intelligent sequential systems: Given that bone metastasis progression and tissue repair are highly dynamic, multi-stage processes, single-payload delivery is inherently insufficient. Next-generation platforms must possess temporally programmable, multi-stage responsive architectures. Ideally, these smart systems should operate sequentially: first releasing matrix metalloproteinase (MMP)-responsive capsular small molecules to inhibit local TGF-β and reverse severe immunosuppression; subsequently exposing an inner core to discharge STING agonists or mRNA payloads to activate cytotoxic T-cell expansion; and finally unlocking bone morphogenetic protein-2 (BMP-2) or osteogenic growth factors to actively repair osteolytic bone defects.
3. Shifting from “cell-targeting” to “intercellular interaction-targeting”: Rather than merely ablating isolated cell populations, future nanomedicines should focus on disrupting crucial structural and metabolic synapses between distinct cell types. Inspired by the target-net-ligand (TNL) systems, future architectures should be designed to physically block or chemically disrupt intercellular connections, such as tumor-osteoclast migrasomes, osteoblast-osteoclast gap junctions, or dendritic cell-Treg tolerogenic synapses, thereby freezing the cellular communication networks that perpetuate the vicious cycle of skeletal destruction.
4. Rational combinations with standard-of-care clinical regimens: To maximize translational viability, nanomedicines must not be developed in isolation but as synergistic adjuvants to existing clinical guidelines. Precise scheduling matrices must be established: coordinating the timing of bone-targeted nanomedicines with bone-modifying agents (e.g., denosumab or zoledronic acid) and immune checkpoint inhibitors (e.g., anti-PD-1/anti-PD-L1 antibodies). For example, deploying nanoplatforms to disrupt the immunosuppressive stromal barrier shortly after denosumab-mediated osteoclast restriction can optimize the therapeutic window, allowing systemically administered anti-PD-1 antibodies to successfully infiltrate and eradicate residual macro-metastases.

## Glossary

BM: bone metastasis
TME: tumor microenvironment
TIME: tumor immune microenvironment
ICB: immune checkpoint blockade
DC: dendritic cell
MDSC: myeloid-derived suppressor cell
TAM: tumor-associated macrophage
Treg: regulatory T cell
Teff: effector T cell
Tpex: precursor exhausted T cell
Tex: exhausted T cell
Tcm: central memory T cell
Tem: effector memory T cell
TRM: tissue-resident memory T cell
NK: natural killer cell
PD-1: programmed cell death protein 1
PD-L1: programmed death-ligand 1
CTLA-4: cytotoxic T-lymphocyte antigen 4
IDO-1: indoleamine 2,3-dioxygenase 1
IFN-γ: interferon gamma
TNF-α: tumor necrosis factor alpha
IL: interleukin
TGF-β: transforming growth factor beta
ECM: extracellular matrix
EMT: epithelial–mesenchymal transition
HSC: hematopoietic stem cell
PEG: polyethylene glycol
PLGA: poly(lactic-co-glycolic acid)
PNPs: polymeric nanoparticles
LNPs: lipid nanoparticles
SLNs: solid lipid nanoparticles
NLCs: nanostructured lipid carriers
PDT: photodynamic therapy
STING: stimulator of interferon genes
DOX: doxorubicin
FOXM1: forkhead box protein M1
GNPs: gold nanoparticles
siRNA: small interfering RNA
TNBC: triple-negative breast cancer
NSCLC: non-small cell lung cancer
